# Madelung Deformity

**Published:** 2016-08-08

**Authors:** Andrew Tranmer, Donald Laub

**Affiliations:** ^a^University of Vermont College of Medicine; Burlington; ^b^University of Vermont Medical Center; Burlington

**Keywords:** Madelung deformity, Vicker's ligament, radial dome osteotomy, Vicker's ligament physiolysis, upper extremity congenital anomaly

## DESCRIPTION

**Figure 1:**
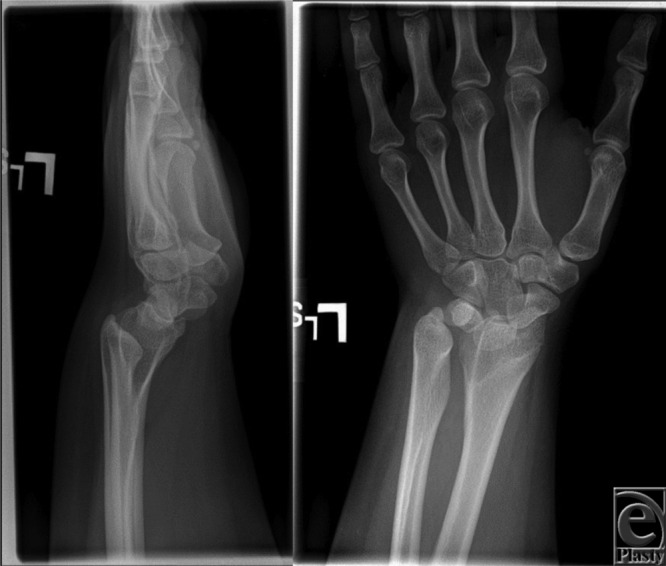
Patient's plain radiographs of the left wrist.

The patient was a 17-year-old adolescent girl who presented with progressive left wrist pain over the past 5 years. Pain was most severe with dorsiflexion. Previously, she had been an active gymnast but worsening wrist pain forced her to stop. Plain radiographs (Fig 1) were consistent with Madelung deformity (MD).

## QUESTIONS

1. **What is the pathogenesis of Madelung deformity?**
2. **What is the clinical presentation of Madelung deformity?**
3. **What are the radiological findings associated with Madelung deformity?**
4. **What are the treatment options for Madelung deformity?**

## DISCUSSION

**Figure 2:**
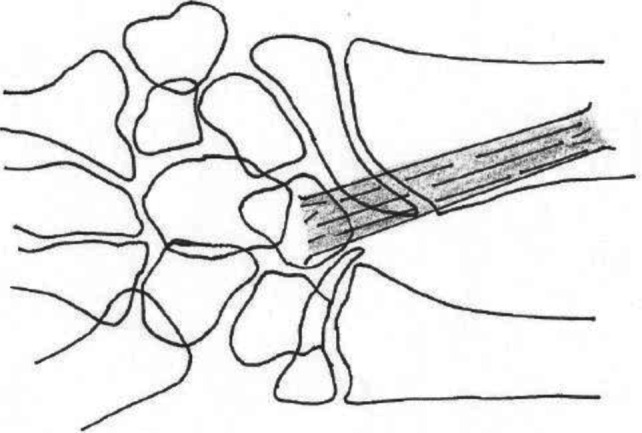
Vicker's (radiolunar) ligament. Drawing by Sophie Mench, DPT.

Madelung deformity is the result of premature growth plate arrest at the medial volar aspect of the distal radius.^2^ Congenital MD can occur as a part of Leri-Weill dyschondrosteosis (LWD) or Turner syndrome.^2^ A Madelung-like deformity can be acquired as the result of repetitive traumatic pressure, commonly in competitive gymnasts. Congenital MD is thought to be a result of a mutation or absence of the short stature homeobox (*SHOX*) gene, which has been identified as a major genetic cause of LWD.^2^ Madelung deformity is associated with a Vicker's ligament, an abnormal volar radiolunar ligament (Fig 2). This abnormal ligament tethers to lunate to the volar distal radius and is hypothesized to limit growth by compressing the epiphyseal plate.^3^

A typical patient will present in early adolescence, most commonly females between the ages of 6 to 13 years. Patients will report disfigurement based on their prominent ulna despite the underlying pathology involving the distal radius. Common presenting complaints include impaired grip strength, limited range of motion, and wrist pain.^2^ The spectrum of presentation is broad and therefore the presenting symptoms are heterogeneous.^2^ For patients who present with suspected MD, wrist radiographs should be obtained for evaluation.

**Figure 3:**
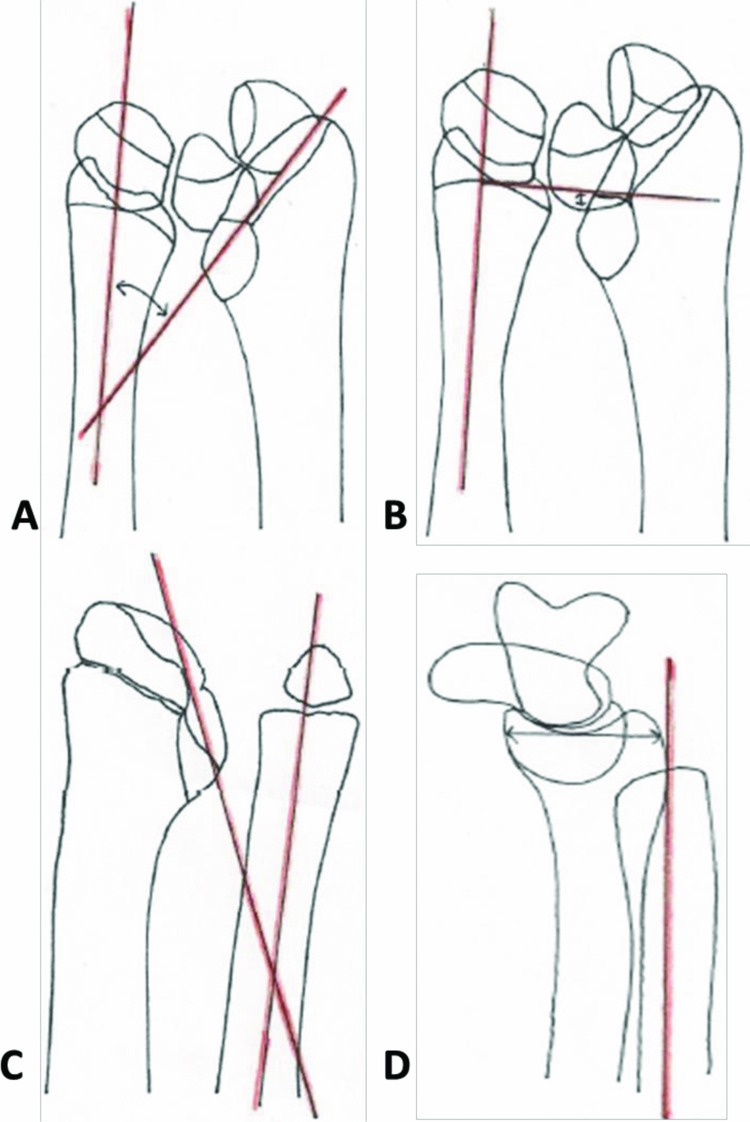
(a) Ulnar tilt as seen on the PA radiograph; calculated as the angle between the long axis of the ulna and line tangential to proximal surfaces of scaphoid and lunate. (b) Lunate subsidence as seen on the PA radiograph; calculated as the distance between the most proximal point of lunate and a line perpendicular to the long axis of the ulna through its distal articular surface. (c) Lunate fossa angle as seen on the PA radiograph; calculated as the angle between the long axis of the ulna and a line through the lunate fossa of the radius. (d) Palmar carpal displacement as seen on the lateral radiograph; calculated as the distance between the long axis of ulna and the most palmar point on the surface of lunate or capitate. Drawings by Sophie Mench, DPT. Descriptions as explained by McCarroll et al.^1^ PA indicates posteroanterior.

Madelung deformity can be diagnosed with lateral and anteroposterior plain radiographs. There is a wide range of anatomical variation in both normal and Madelung wrists. Specific thresholds have been determined to most accurately detect MD. These limits are at least 33° of ulnar tilt, at least 4 mm of lunate subsidence, lunate fossa angle of 40° or more, and 20 mm or more of palmar carpal dispalcement^1^^,^^4^ (Fig 3). A spectrum of MD may exist, but it is unclear if milder forms of the disease, based on symptoms or radiographic findings, are clinically significant; they may or may not progress to bona fide, severe MD.^4^ We are not currently able to accurately predict the natural course of disease.

If patient symptoms are severe and radiological evidence supports MD, surgical intervention may be undertaken—although there is no universally preferred surgical strategy. Operative procedures can target the radius deformity, the ulnar protuberance, the abnormal Vicker's ligament, or a combination of the 3. Today's standard of care is a radial dome osteotomy with Vicker's ligament physiolysis/release. Two trials showed that this procedure resulted in decreased pain, improved cosmetic appearance, and increased range of motion while preserving the distal radioulnar joint.^5^^,^^6^ Physiolysis must be performed in a small and distinct area as to not sacrifice support of the lunate.^3^ Steinman et al^6^ recommend waiting until skeletal maturity to proceed with intervention because pain symptoms may resolve over time. Ulnar surgery may be undertaken in severe cases; proposed criteria for ulnar surgery include increased lunate subsidence, ulnar variance, and palmar carpal displacement. This may be required to achieve a more appropriate length symmetry between the radius and ulna in patients with significant deformities.^7^ New technologies are being developed with 3-dimensional reconstructions to help visualize and plan osteotomies to provide improved patient outcomes; these methods, however, remain costly, time-consuming, and not widely available.^8^

In summary, MD is a rare pediatric and adolescent condition caused by premature growth arrest along the medial volar aspect of the distal radius. Patients will commonly present with wrist pain and limited range of motion. There are unique radiographic deformities that can be seen on plan radiographs that help confirm diagnosis. Surgery may be undertaken if pain is severe and functionality is poor. Although there are numerous proposed surgical techniques, the currently preferred method involves distal radial dome osteotomy with Vicker's ligament release. Surgery has been shown to decrease pain, increase range of motion, and improve the aesthetic appearance of the wrist.
